# Optical orbital-angular-momentum-multiplexed data transmission under high scattering

**DOI:** 10.1038/s41377-019-0140-3

**Published:** 2019-03-06

**Authors:** Lei Gong, Qian Zhao, Hao Zhang, Xin-Yao Hu, Kun Huang, Jia-Miao Yang, Yin-Mei Li

**Affiliations:** 1 Department of Optics and Optical Engineering, University of Science and Technology of China, Hefei, 230026 China; 2 Andrew and Peggy Cherng Department of Medical Engineering, Department of Electrical Engineering, California Institute of Technology, Pasadena, CA 91125 USA; 3 Hefei National Laboratory for Physical Sciences at the Microscale, University of Science and Technology of China, Hefei, 230026 China

**Keywords:** Fibre optics and optical communications, Imaging and sensing

## Abstract

Multiplexing multiple orbital angular momentum (OAM) channels enables high-capacity optical communication. However, optical scattering from ambient microparticles in the atmosphere or mode coupling in optical fibers significantly decreases the orthogonality between OAM channels for demultiplexing and eventually increases crosstalk in communication. Here, we propose a novel scattering-matrix-assisted retrieval technique (SMART) to demultiplex OAM channels from highly scattered optical fields and achieve an experimental crosstalk of –13.8 dB in the parallel sorting of 24 OAM channels after passing through a scattering medium. The SMART is implemented in a self-built data transmission system that employs a digital micromirror device to encode OAM channels and realize reference-free calibration simultaneously, thereby enabling a high tolerance to misalignment. We successfully demonstrate high-fidelity transmission of both gray and color images under scattering conditions at an error rate of <0.08%. This technique might open the door to high-performance optical communication in turbulent environments.

## Recovering scattered data from twisted light

Twisted light beams can be made to transmit higher quality data by decoding the information present in the ‘speckle patterns’ that arise when they pass through scattering media. Lei Gong of the University of Science and Technology of China and colleagues developed the ‘scattering-matrix-assisted retrieval technique’ (SMART) to recover scattered data from multiplexed multiple orbital angular momentum (OAM) channels. These multiple twisting light beams have the potential to carry unlimited data channels, but light scattering, caused by micro-particles in the atmosphere or by energy transfer between channels, reduces data quality. The SMART platform allowed high-fidelity transmission of images, reducing the error rate by 21 times compared to previous reports. Improvements on the technique could facilitate the transfer of high quality optical data in harsh atmospheric or underwater conditions.

## Introduction

Light is one of the main carriers of information in communication. Fully enhancing the information-carrying capacity and spectral efficiency of light has always been a perennial goal in both academia and industry, and it is traditionally realized by multiplexing the wavelength^1^, polarization^2^, and spatial degree of freedom^3^ of light to increase the data channels for a parallel transformation. Recently, the orbital angular momentum (OAM) of light has been considered as a promising degree of freedom for multiplexing data in free space^4–6^ and optical fibers^7,8^ and at the nanoscale^9–13^. The OAM of light was recognized by Les Allen in 1992^14^. A light beam carrying OAM possesses a helical wavefront, described by exp(*ilϕ*), where *ϕ* indicates the azimuthal angle, and the topological charge *l* is an unbounded integer. Superior to spin angular momentum (i.e., circular polarization) with two states, OAM could offer unlimited channels for data transmission. Due to this unique property, OAM multiplexing has been widely applied to achieve high-capacity communication in free space^15–17^ and optical fibers^7,8^. However, optical free-space communication using OAM multiplexing inevitably suffers from the issue of multiple scattering from ambient microparticles in the atmosphere^18–20^, which will scramble the wavefronts of the OAM modes and destroy the orthogonality between the OAM channels^18,21^. In OAM-multiplexing technology, the deteriorated orthogonality will increase the crosstalk when the information carried in different OAM channels is demultiplexed^4,5,15,22^. Consequently, it is challenging to realize OAM-based communication through scattering media. Apart from scattering media, due to mode coupling and dispersion, optical transmission through multimode systems including fibers^23,24^ and waveguides^21^ also suffers from similar problems.

The propagation of light beams through scattering media or multimode systems yields well-known speckle patterns that arise from the self-interference of multiply scrambled light^25–27^. Although these speckle patterns are different from the incident patterns, the encoded information is still contained in the speckles and is never lost. Actually, the speckle patterns depend on the temporal and spatial properties of the incident light, which enable the extraction and utilization of the information in the speckles. For example, in a time-invariant complex medium, the temporal variations of a speckle pattern can be harnessed to resolve the wavelengths of a light source, which is the fundamental origin of a speckle wavemeter or spectrometer with subfemtometer resolution over a wide operating spectrum^28^. In the spatial domain, multiple light scattering can be used in depth-tissue imaging^25,29^, three-dimensional (3D) imaging^30–32^ and displays^33^, scattered material recognition^34^, and spatial coherence measurements^35^.

Here, we propose a scattering-matrix-assisted retrieval technique (SMART) to precisely extract encoded OAM states from multiply scattered light. This SMART first employs a speckle-correlation scattering matrix to recover the optical field of a data-carrying vortex beam with OAM superposition states and then demultiplexes every OAM channel with the mode decomposition method. To test its validity, we have built an optical wireless data transmission system in which a digital micromirror device (DMD) is used to encode parallel OAM channels and realize reference-free calibration in a multiple scattering environment. Notably, the SMART has a good tolerance to system misalignment and permits a non-line-of-sight (NLOS) connection for use in optical communication. After undergoing multiple scattering, the data-carrying vortex beam generates a random speckle pattern, which is recorded by a camera and then analyzed with our SMART platform. Experimentally, we achieve a low crosstalk of –13.8 dB in the parallel sorting of 24 scattered OAM channels and demonstrate high-fidelity transmission of both gray and color images at a low error rate of <0.08%, which is enhanced by ~ 21 times compared with previous reports^36^.

## Results

### Principle of the SMART

**Fig. 1:**
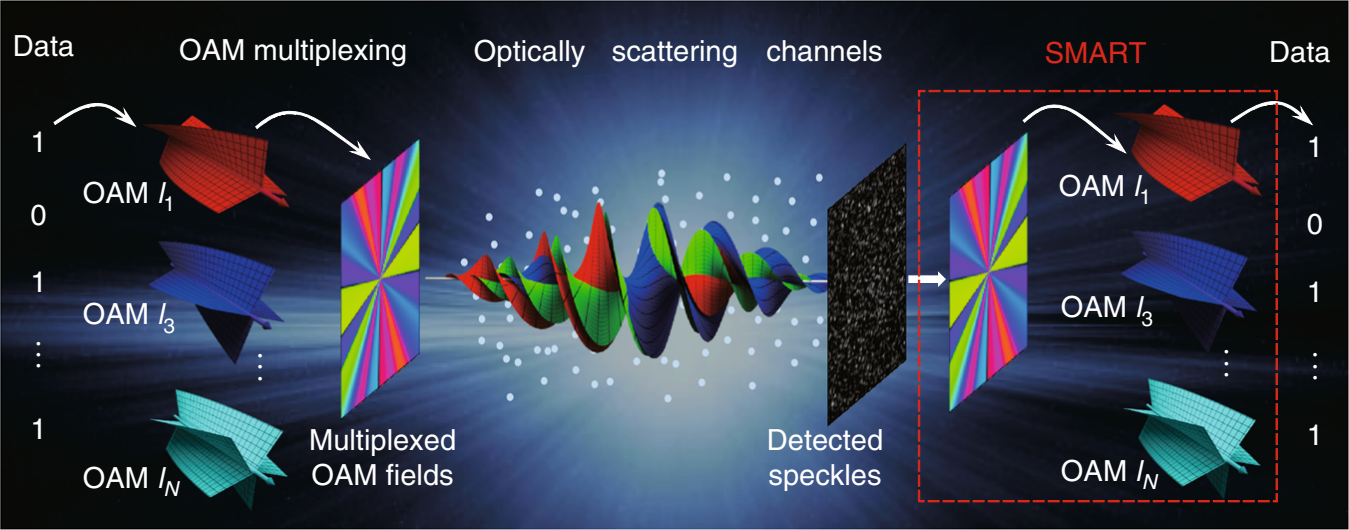
Concept of SMART-enabled OAM-multiplexed transmission across scattering channels. Information is encoded into an OAM superposition state. A data-carrying vortex beam propagates across scattering channels. At the receiver, the SMART retrieves the original field from the scattered random speckles and completes OAM demultiplexing from the retrieved field. On this basis, the data carried by light can be extracted from the reconstructed OAM spectrum

Figure 1 illustrates a conceptual diagram of OAM-multiplexed data transmission under multiple scattering. In the transferred data, N-bit data (with the *n*-th bit taking a binary value of *c*_*n*_ = 0 or 1) are encoded into an optical OAM superposition mode $E_S = \mathop {\sum}\nolimits_{l = l_1}^{N_l} {c_nE_{l_n}}$, where $n = 1,2, \ldots ,N_l$, and the OAM eigenstate $E_{l_n} = A_n\mathrm{exp}\left( { - il_n\phi } \right)$ corresponds to the data channel of the *n*-th bit. After undergoing multiple scattering, these independent OAM channels are scrambled and mixed so that only the speckle pattern can be detected at the receiver. To extract the data from the speckle pattern, all the OAM channels must be identified precisely. To realize this identification, our SMART platform first recovers the incident field based on the speckle-correlation scattering matrix and then demultiplexes the OAM channels from the retrieved field by using spatial mode decomposition. Thus, we can decode the transferred data {*c*_*n*_} by addressing all OAM channels, which implies that optical OAM-multiplexed data transmission through optically scattering channels is feasible.

The underlying principle governing the SMART is that multiple scattering can be taken as a linear and deterministic process in mathematics

1 $$y = {\mathbf{T}}x$$

where **T** is the transmission matrix (TM, an *M* × *N* matrix) of the scattering channels and correlates the input field *x* (an *N* × 1 vector) with the output field *y* (an *M* × 1 vector). The *p*-th column of the TM, *t*_*p*_, is the scattered field corresponding to the input field *k*_*p*_, one of *N* orthonormal bases $k_1,k_2, \ldots ,k_N$. For an arbitrary incident field $x = \mathop {\sum}\nolimits_{p = 1}^N {\alpha _pk_p}$, the resultant field can be expressed as $y = \mathop {\sum}\nolimits_{p = 1}^N {\alpha _pt_p}$, where $\alpha _1,\alpha _2, \ldots ,\alpha _N$ are complex coefficients that contain the information of the incident field. Thus, if the complex-valued TM is calibrated or given, one can retrieve any incident field from its output field. Usually, measuring such a complex field requires a highly stable interferometric setup, which is undesired in free-space communication links. To solve this problem, we propose a reference-free calibration technique based on a DMD and develop its corresponding calibration algorithm (see Methods).

Based on a calibrated TM, the speckle-correlation scattering matrix^32^ can directly associate the incident field with the resultant intensity $y^{\ast}y$, enabling the direct retrieval of the incident field from a single-shot recording of the speckle pattern. The scattering matrix reads^32^

2 $${\mathbf{Z}}_{pq} = \frac{1}{{{\mathrm{\Sigma }}_p{\mathrm{\Sigma }}_q}}\left[ {t_p^ \ast t_qy_p^ \ast y_{qr} - t_p^ \ast t_{qr}y_p^ \ast y_{qr}} \right]$$

where $\cdot _r$ indicates a spatial average, $\sum_p = \vert{{t}_{p}}{\vert^{2}_{r}}$ and the symbol $^{\ast}$ denotes the complex conjugate of the corresponding variable. Under the assumption that $t_1,t_2, \ldots ,t_N$, and *y* are randomly diffused fields due to high scattering, the TM can be considered as a Gaussian random matrix. Then, Eq. (2) is rewritten as

3 $${\mathbf{Z}}_{pq} = \alpha _p\alpha _q^ \ast + \frac{1}{{{\mathrm{\Sigma }}_p{\mathrm{\Sigma }}_q}}t_p^ \ast y^ \ast _rt_qy_r$$

where $\alpha _p = \frac{1}{{{\mathrm{\Sigma }}_p}}t_p^ \ast y_r$. Since the columns of the TM are uncorrelated to each other, the general orthogonality relations $\frac{1}{{\sqrt {{\mathrm{\Sigma }}_p{\mathrm{\Sigma }}_q} }}t_p^ \ast t_{qr} = \delta _{pq}$ and $\frac{1}{{{\mathrm{\Sigma }}_p}}t_py_r = 0$ hold. Thus, the scattering matrix becomes ${\mathbf{Z}}_{pq} = \alpha _p\alpha _q^ \ast$, the sole eigenvector of which is the incident field. In practice, the second term in Eq. (3) can be negligible if the number of sampled output modes is much larger than the number of preset input bases.

Once the incident field is retrieved, OAM sorting can be performed by applying spatial mode decomposition^37^. For instance, when the data-carrying OAM field *E*_*S*_ is precisely recovered, the complex-valued coefficients *c*_*n*_ can be calculated by

4 $$c_n = {\int\!\!\!\!\!\int} {E_SE_{l_n}^ \ast rdrd\phi }$$

Then, the probability *P*(*l*_*n*_) of finding a photon in an *l*_*n*_-order OAM state is defined as

5 $$P\left( {l_n} \right) = \frac{{\left| {c_n} \right|^2}}{{\mathop {\sum }\nolimits_{n = 1}^{N_l} \left| {c_n} \right|^2}}$$

which can also be referred to as the OAM power spectrum^37^. Thus, the SMART allows us to accurately characterize the OAM components from multiply scattered light, thereby enabling data transmission.

### Experimental setup and characterization

**Fig. 2:**
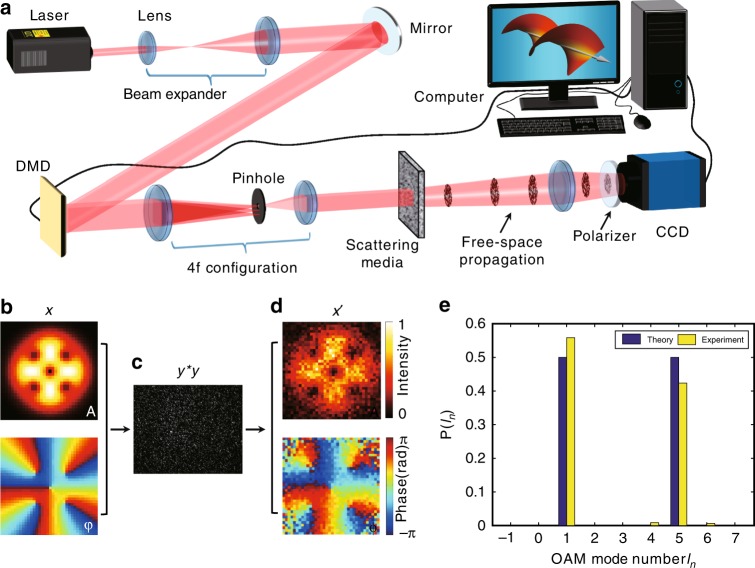
Experimental setup and characterization of the SMART platform. **a** Experimental setup of the SMART platform. **b**–**d** Field retrieval of a known incident field. For a given LG superposition field (*x*; (**b**)), a raw intensity speckle with a single shot (y*y; (**c**)) is recorded. The retrieved field (*x’*; (**d**)) is achieved by using the SMART. The symbols *A* and *φ* denote the amplitude and phase of the fields, respectively. **e** A comparison between the measured OAM spectrum by the SMART and the theoretical spectrum

To validate the SMART platform experimentally, we built an optical data transmission link based on a DMD, as illustrated in Fig. 2a (see more details in Methods). Due to the capability of complex field modulation and a high refresh rate, a single DMD enables high-fidelity generation of OAM fields and rapid switching among them (see Methods). An optical diffuser (DG10–220, Thorlabs, Inc.) is employed to mimic an optically scattering environment and inserted in the transmission path. A TM calibration of the scattering channels is required before carrying out optical transmission. Here, we introduce a technique developed from the parallel wavefront optimization method^38,39^ to achieve rapid reference-free calibration in the same setup (see Methods). By modulating the wavefront of the incident light, the TM, a collection of diffused fields for all input modes, can be derived from the transmitted speckle patterns with the calibration algorithm, which is described in detail in Supplementary Note 1. In this work, the experimentally calibrated TM can be found in Supplementary Fig. 1.

As a proof-of-concept demonstration, the SMART with a calibrated TM is used to recover a known light field of a superimposed Laguerre-Gaussian (${\mathrm{LG}}_p^l$) mode (${\mathrm{LG}}_0^1$ and ${\mathrm{LG}}_0^5$, see Fig. 2b), which is created by the DMD. After the light passes through a scattering medium, the **Z**-matrix is calculated by substituting the calibrated TM and the recorded speckle pattern ($y^{\ast}y$, see Fig. 2c) into Eq. (2). Finally, the only eigenvector (*x*′) of the **Z**-matrix is the retrieved incident field, whose amplitude and phase profiles are shown in Fig. 2d. The incident field is well recovered with some ignorable speckle noise, so its OAM power spectrum can be calculated with high accuracy, as shown in Fig. 2e. Good coincidence between the theoretical and experimental results is achieved with inevitable deviations, which is attributed to environmental noise. In addition, the relative sampling rate *γ = M/N* also affects the accuracy of the field retrieval, where *M* (e.g., 480 × 640 in our case) and *N* (fixed to 36 × 36 in this work) are the numbers of sampled points in the output field and input field, respectively. Note that a large signal-to-noise ratio (SNR) and a high sampling rate can enhance the accuracy of the field retrieval, as shown in Supplementary Fig. 2.

**Fig. 3:**
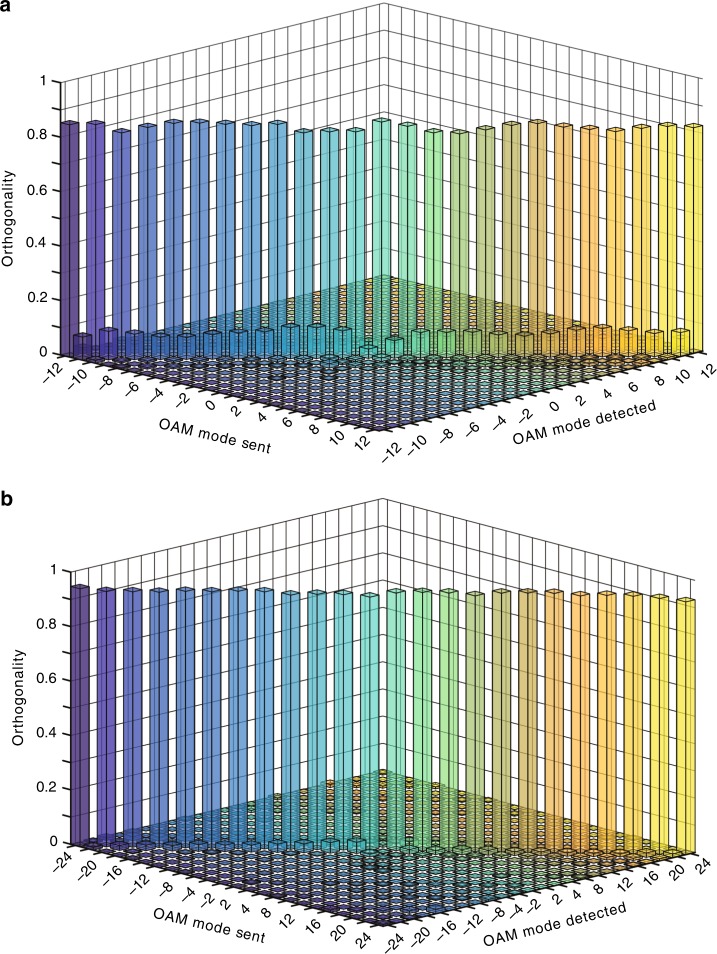
Measured orthogonality relations between the scattered OAM states. **a** The measured coincidence between OAM states with their topological charges from *l*_*n*_ = –12 to *l*_*n*_ *=* 12 at an interval of 1. The maximum crosstalk is −9.4 dB. **b** The measured coincidence for another OAM base (*l*_*n*_ = –24, –22, ···, 24) with a state interval of 2. The maximum crosstalk is –13.8 dB

In OAM-multiplexing technology, the orthogonality between OAM channels guarantees efficient (de)multiplexing. For our SMART platform, we examine the orthogonality between the recovered OAM channels after undergoing multiple scattering. Experimentally, we measure the intermodal crosstalk (Supplementary Note 2) of OAM states with topological charges from *l*_*n*_ = –12 to *l*_*n*_ = 12 with a step size of 1. For each input mode, the measured orthogonality between the retrieved mode and all the sent OAM bases is shown in Fig. 3a, where a maximum crosstalk of –9.4 dB is achieved. To further decrease the crosstalk, we employ another OAM base with a state interval of 2 (e.g., *l*_*n*_ = –24, –22, ···, 24). Figure 3b displays the measured crosstalk with a maximum value of –13.8 dB. This implies that a large interval between two adjacent bases leads to a low crosstalk, which has also been observed in quantum entanglement between rational-order OAMs^40^. Such a low crosstalk at a level of –13.8 dB is acceptable for practical usage^22^, and OAM bases with an interval of 2 are employed in the following experiments.

In addition, optical free-space communication usually relies on a line-of-sight connection between the transmitter and receiver (Supplementary Fig. 3a), implying a requirement of rigorous alignment in the optical system. In contrast, our technique has a good tolerance to system misalignment and even permits a NLOS connection (Supplementary Fig. 3b) because the SMART can retrieve the incident field from one part of the received speckle pattern, which works as traditional holograms. In our NLOS experiment with a tilting deviation of ~4 degrees (the angle between the optical axis of the collecting lens and that of the optical vortex), we can still achieve a crosstalk as low as –12.5 dB (Supplementary Fig. 4), which has no significant difference compared with –13.8 dB in the well-aligned configuration. This result demonstrates that our SMART platform is robust in its experimental implementation due to an immunity to misalignment, which is advantageous for transferring data in practical applications.

### Optical data transfer under scattering

**Fig. 4:**
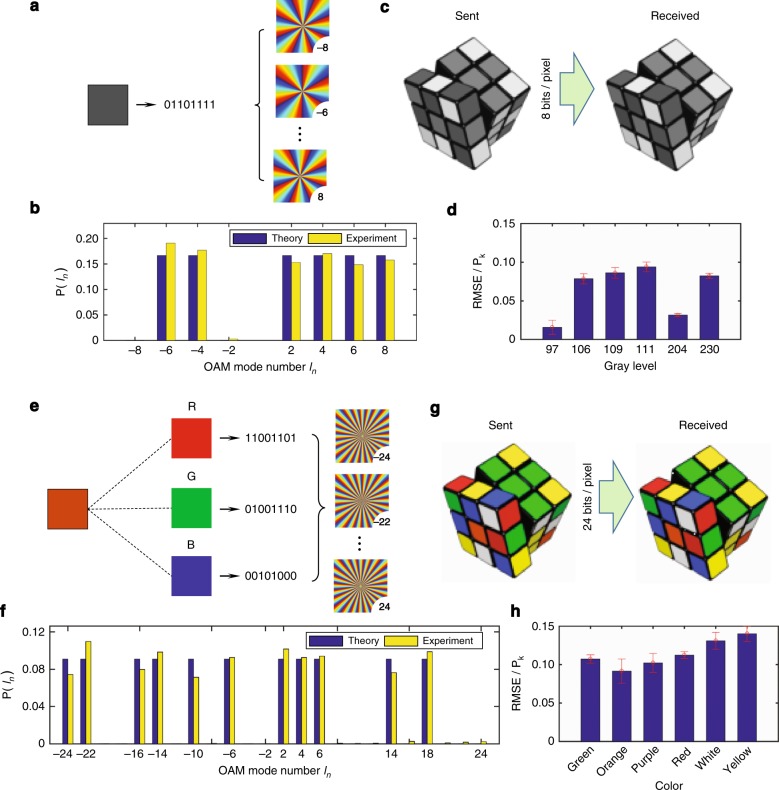
Results of OAM-encoded data transfer through a scattering medium. **a** Gray-level encoding scheme using 8-OAM multiplexing for transferring grayscale images. **b** Theoretical and experimental OAM spectra of the gray level 111. **c** Example of sent and received grayscale images (Rubik’s Cube, 100 × 100 pixels) in a data transmission experiment. The Rubik’s Cube^®^ was used with the permission of Rubik’s Brand Ltd (www.rubiks.com). An error rate of 0 was achieved for this image transmission. **d** The relative error RMSE/*P*_*K*_ of all gray levels contained in the picture in (**c**). **e** RGB encoding scheme using 24-OAM multiplexing, applied to color images. **f**, **g** The corresponding results for optical transfer of a color image of a Rubik’s Cube. An error rate of 0.08% was achieved for the color image data transfer. **h** The relative error RMSE/*P*_*K*_ of all colors contained in the picture in (**g**)

For the purpose of communication, the binary data carried in multiplexed OAM states are encoded into a single laser beam. In a grayscale picture, 256 gray levels can be represented by using a binary digital byte with 8 bits, where every bit takes a value of 0 or 1. To encode the byte, we use an OAM superposition state containing 8 OAM bases (e.g., the topological charges *l*_*n*_ = ±8, ±6, ±4, and ±2 in Fig. 4a), each of which correlates with one bit. For one bit, the value 1 (or 0) indicates that its corresponding OAM component has a spectral value of *P*_*K*_ (or 0) in the superposition state, where *P*_*K*_ = 1/*K*, and *K* is the number of 1-value bits in a byte. For example, the gray level of 111 in Fig. 4b has the binary byte of ‘01101111’, where *K* = 6 and its theoretical *P*_*K*_ = 1/6 in the OAM spectrum. For data transfer experiments, information encoding is realized by directly generating a light field representing the OAM superposition state. By using our SMART platform, the retrieved OAM spectrum is also provided in Fig. 4b, which shows good agreement with the theoretical result. For OAM-encoded data extraction, the retrieved spectrum can be converted into the received data by using a simple criterion: the *n*-th bit takes the value of 0 if its measured *P*(*l*_*n*_) < *P*_*K*_/2 or 1 if *P*(*l*_*n*_) ≥ *P*_*K*_/2. In fact, the value of *P*_*K*_ changes with the number of 1-value bits. In our experiment, we normalize the spectral values to the maximum value so that *K* is evaluated by the number of OAM channels with normalized values larger than 1/2.

By following this strategy, we attempt to transfer a gray picture (Rubik’s Cube, the left panel in Fig. 4c) with 100 × 100 pixels through a scattering medium. Experimentally, we can receive its transferred picture (see the right panel in Fig. 4c) with an error rate of 0, which is defined as the ratio of incorrect pixels in the decoded image to all the pixels of the image^36^. This indicates that every pixel in the picture has been transferred and retrieved in a nearly perfect way. Such a high performance arises from the low error of every OAM channel in the retrieved spectrum. Note that the error rate of transferring pictures is obtained after numerically correcting the real data error. To validate the error quantitatively, we show the root-mean-square errors (RMSEs) between the theoretical and measured OAM spectra in Fig. 4d by addressing all gray levels contained in the picture. The ${\mathrm{RMSE}} = \sqrt {\mathop {\sum }\limits_{n = 1}^{N_l} \left[ {P_{\mathrm{Exp}}\left( {l_n} \right) - P_{\mathrm{Theory}}\left( {l_n} \right)} \right]^2{\mathrm{/}}N_l}$, where *N*_*l*_ = 8 for an 8-bit gray level. Figure 4d indicates that the RMSE is located at the level below 0.1*P*_*K*_, which is much smaller than the threshold 0.5*P*_*K*_ in our criterion and therefore promises high-fidelity data transmission. A detailed comparison between the theoretical and experimental OAM spectra for more gray levels can be found in Supplementary Fig. 5.

To further transfer a color image, we employ a superposition state of 24 OAM components (i.e., *l*_*n*_ = ±24, ±22, …, ±4 and ±2) to encode the data. Any color can be expressed by a weighted mixture of three primary colors (i.e., red, green, and blue), as shown in Fig. 4e. These 24 OAM bases are divided into three groups, which correlate with the three primary colors. For one primary color, 256-level data are encoded into eight OAM components (i.e., 8 bits), which works as in the case of the grayscale image. Thus, an RGB color can be transferred by using one-time data transmission with this OAM superposition state. Figure 4f shows the theoretical and experimental OAM spectra during the transmission of an ‘orange’ pixel. The good agreement between the spectra indicates that our SMART platform also behaves well for data transmission with more OAM channels. More examples of transferring other colors can be found in Supplementary Fig. 6. Based on these results, we have transferred a color image of a Rubik’s Cube (see Fig. 4g) and received it with an error rate of 0.08%, which is enhanced by ~21 times compared with 1.7% in ref. ^27^. The RMSEs (where *N*_*l*_ = 24) of these colors in this picture are plotted in Fig. 4h, which shows a larger error than the case of a gray-level picture. Due to the large *K* in 24 bits, *P*_*K*_ decreases so that the relative error RMSE/*P*_*K*_ increases. Nevertheless, the error is still much smaller than the threshold criterion, promising a low error in data transmission.

Moreover, a theoretical investigation of the transmission accuracy (see Supplementary Note 3) of our SMART platform has been implemented under different SNRs and sampling parameters *γ*. The simulated results in Supplementary Fig. 7 reveal that the transmission accuracy is better than 99.66% when γ ≥ 25 and SNR > 2. This theoretical prediction agrees well with our achieved results (with error rates below 0.08%), where γ = 237 and SNR ≈ 4 (estimated by using the RMSEs in Fig. 4d, h) in our experiments.

## Discussion

**Fig. 5:**
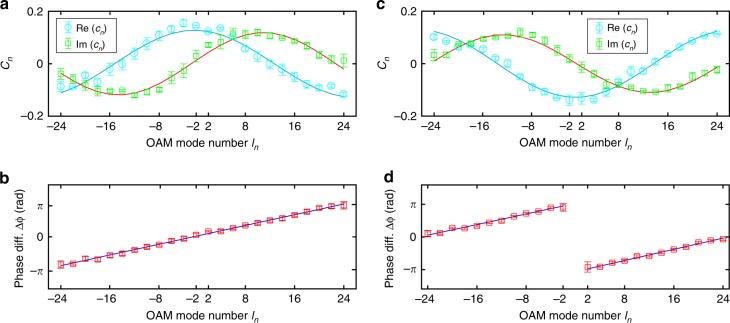
Measurement of the complex coefficients and phase shifts of OAM components. **a**, **b** The real (blue circles) and imaginary parts (green squares) of the measured OAM coefficients with an *l*_*n*_-dependent phase $\phi \left( {l_n} \right) = \pi l_n{\mathrm{/}}24 + \phi _0$, with preset phase shifts of *ϕ*_0_ = 0 (**a**) and π (**b**). The theoretical data are plotted as blue and red lines. **c**, **d** The corresponding phase difference (diff.) $\Delta \phi \left( {l_n} \right)$ between the calculated phase $\phi \left( {l_n} \right)$ plotted as a function of *l*_*n*_. Linear fitting (solid lines) to the phase difference is calculated. The error bars are calculated as the standard error of 20 measurements

In addition to transferring binary digital data, our SMART platform can also measure the phase shifts of OAM components, since the complex coefficients *c*_*n*_ of each OAM base can be retrieved from the random speckles. To demonstrate this measurement, we use a superposition state comprising 24 OAM components with an *l*_*n*_-dependent phase $\phi \left( {l_n} \right) = \pi l_n{\mathrm{/}}24 + \phi _0$, where *ϕ*_0_ controls the phase shift. In the SMART, we only need to retrieve the incident field and extract the coefficients *c*_*n*_ by using the mode composition method, without the requirement of calculating the OAM spectrum. Figure 5a, b presents the results for the two tested states with phase shifts of *ϕ*_0_ = 0, π, respectively. Using these quantities, the relative phase differences between the OAM components are calculated and plotted in Fig. 5c, d. We can observe a distinct *l*_*n*_-dependent phase ramp with a fitting slope of 0.259 ± 0.002 rad per mode (Fig. 5c), which matches well with the theoretically predicted value of 0.262. As expected in Fig. 5d, a π-phase jump appears at *l*_*n*_ = ±2 for the tested state with a preset phase shift of π. Thus, our SMART platform exhibits great potential for complex spectral analysis and the measurement of phase.

Compared with previous OAM-demultiplexed systems^6–8,10,41^, our SMART has two differences. First, in traditional systems, OAM channels are physically projected onto different spatial locations for the purpose of discrimination. Our SMART employs a digital method to distinguish every OAM channel with the help of a computer. Second, traditional systems directly demultiplex every channel from an OAM superposition state in free space. Our SMART needs to initially recover the OAM superposition state from highly scattered speckles and then demultiplex every OAM channel. The implementation of the SMART involves precalibration and data post processing, which is performed at the cost of resources and time compared with existing demultiplexing techniques.

Additionally, it should be noted that OAM-based data transmission operates over a distance of ~3 m in a laboratory environment, and the data analysis is carried out on a personal computer (see Methods for specifications). In practical optical communication, due to increased scattering events, the SNR will decrease as the transmission distance increases, which will deteriorate the performance of the SMART in communication. A higher power laser, a larger aperture collecting lens, and good alignment in the optical system can improve the SNR for long-distance transmission. In principle, if the speckles can be measured and the TM calibration is still available, the number of scattering events does not affect the validity of the SMART. Due to its robustness, our SMART platform could work well in an urban environment where atmospheric fluctuations occur at fast time scales. To calibrate the TM in real time, the hardware in the SMART needs to be updated. In our current system, calibration can be achieved within 0.22 s when the switching rate of the DMD is fully used (see Methods). Therefore, a faster DMD and a lock-in camera^42^ can be utilized to accelerate the calibration process. Furthermore, our method can be readily implemented with a multimode fiber system, which could be well suited for ultrahigh capacity optical transmission in the future.

Data transfer speed is a key factor in communication. Currently, data are transferred through up to 24-multiplexed OAM channels via the SMART platform. The transfer speed can be further improved by multiplexing more OAM channels at the expense of a lower SNR. For this purpose, a larger number (*N*) of sampled points in the input plane would be better for accurately encoding the light fields, and more laser power is also required to improve the SNR. This SMART platform could also be extended to data transmission with other types of orthogonal modes, such as Hermite-Gaussian beams^43^ and vector beams^44^. Moreover, combining existing frequency-encoding techniques such as quadrature amplitude modulation (QAM)^15^ and quadrature phase shift keying (QPSK)^45^ might allow for high-speed practical communications. Integrating polarization- and wavelength-division multiplexing techniques might be a promising approach to further enhance the information capacity.

As a prototype, our current SMART platform requires updates in three aspects before it is suitable for practical applications. First, a faster DMD and complementary metal oxide semiconductor (CMOS) camera should be equipped to realize real-time calibration. Second, an efficient algorithm to simplify the procedures during data postprocessing must be developed to speed up the retrieval of data. Finally, it is feasible to increase the computing speed by using high-performance computers or cloud computing.

In conclusion, we have proposed and implemented a novel SMART platform for parallel sorting of OAM states from highly scattered speckles and further applied the technique to demonstrate OAM-based transmission through scattering media. In particular, the SMART has a good tolerance to system misalignment and enables a NLOS connection for data transmission. Based on a self-built system capable of channel encoding and reference-free calibration, we achieved an experimental crosstalk as low as –13.8 dB among 24 scattered OAM channels. Furthermore, high-fidelity transmission of both gray and color images at an error rate of <0.08% was achieved. Our technique offers opportunities for high-performance optical wireless communication under scattering conditions, multimode fiber-optic communication^46^, and harsh underwater optical communication^47^. In addition, our results might benefit OAM-based quantum communication^48^, such as high-dimensional quantum key distribution^49^, quantum encryption^50,51^, and quantum memory^52^, in turbulent environments.

## Materials and methods

### Details of the experimental setup

A He–Ne laser (632.8 nm wavelength; Coherent, 31-2140-000) with a power of 35 mW was used as the light source. A beam expander with a magnification of 20 was used to tune the size of the laser beam. The enlarged beam was then steered to fully illuminate the surface of a DMD (1920 × 1080-pixel resolution; ALP 4395, ViALUX GmbH) with an incident angle of 24°. With a 4-*f* configuration and a pinhole filter, the DMD can generate and rapidly switch among OAM beams by complex field encoding. For optical transmission, the data-encoded light fields were transferred over ~3 m in free space. To mimic an optically scattering environment, an optical diffuser (DG10–220, Thorlabs, Inc.) with a large angular distribution of scattering (Supplementary Fig. 8) was inserted into the transmission path. To improve the energy efficiency, a collimating lens (not shown in Fig. 2a) could be introduced behind the diffuser to adjust the divergence angle. At the receiver, a converging lens (*f* = 100 mm) collected the scattered light for field imaging and a CMOS camera (PL-D752MU, PixeLINK) with a polarizer recorded the intensity speckle patterns. Corresponding to high-speed mode switching, synchronous image acquisition was realized based on the trigger output of the DMD controlled by a computer. The computer was also used to execute the calculations in the SMART.

### Generation of OAM beams by a DMD

A full set of OAM modes and multiplexed modes can be generated by a binary DMD. To generate these modes, spatially encoding the amplitude and phase of a light beam using binary holograms is required. We employed a superpixel encoding method^53,54^ to design the required holograms. In this method, the square regions of nearby pixels (4 × 4 pixels within 1080 × 1080 pixels in our case) were grouped into various superpixels to define a complex field in the imaging plane using the first-order diffraction beam. Specifically, according to the phase and normalized amplitude (Supplementary Fig. 9a, b) of each OAM field, a binary hologram was calculated by the superpixel encoding. Supplementary Fig. 9c shows a typical binary hologram, where white and black represent ON and OFF states, respectively. Once the hologram was loaded onto the DMD, the desired OAM field was produced in the imaging plane. The high fidelity of this method enables accurate field generation. Supplementary Fig. 9d, e presents the measured intensity and phase of the generated field on a given plane, which agree with the theoretical distribution. In this manner, other OAM states could be created with their corresponding holograms projected. Furthermore, the high-speed DMD allows us to switch among various OAM states with a rate up to 17.86 kHz^55^ (Supplementary Fig. 9f).

### Reference-free TM calibration

The TM calibration algorithm is developed from the parallel wavefront optimization method^38^, which is elaborated in Supplementary Note 1. To achieve reference-free calibration, the DMD pixels were divided into two groups that could be independently controlled to modulate the signal and reference light. Each segment, i.e., each input mode, in the signal part was assigned with a distinct phase shifting frequency. Thus, the phase of each segment dithers at a unique frequency during the measurement process. This multidithering modulation allowed us to access the complex TM via a Fourier transform (Supplementary Eq. 3). This method does not require an external reference arm and fully uses the pixel resolution of the DMD.

Four steps are required to complete the calibration process. First, one group modulates the signal field, while the other part acts as a reference field (Supplementary Fig. 10a). The second step involves the same procedure as first step but with the roles of the groups exchanged, as illustrated in Supplementary Fig. 10b. After that, two parts of the TM are obtained but with different reference terms (Supplementary Eq. 4). To access the exact TM, however, we need to know the reference field. Therefore, the third step is to perform reference phase matching (Supplementary Eq. 6). To this end, the phase dithering of each group (Supplementary Fig. 10c) rather than each pixel is performed in this step. The last step involves the normalization of the reference intensity, which is achieved by taking one additional image after turning off the signal part (Supplementary Fig. 10d). Finally, we obtain the calibrated TM multiplied by a phase factor of the complex conjugate of the reference field. Nevertheless, this phase term can be dismissed because it does not influence the field retrieval with the **Z**-matrix calculation.

Experimentally, we performed the TM calibration with the same setup used for optical transmission. Multidithering phase modulation was implemented by directly generating the corresponding optical fields with binary holograms calculated with the superpixel method^53,54^. The total number of holograms used for the calibration was determined by the number of input orthonormal bases (*N* = 1296). For each base, three holograms corresponding to three-step phase shifting were used. Considering the additional reference field measurement, a total of 3*N* + 7 patterns are required. The high-speed switching ability of the DMD enables fast calibration. In the experiment, the calibration process took ~22 s using 3895 binary patterns. Currently, the calibration time is mainly limited by the maximum frame rate (180 Hz) of the camera and can be further shortened to 0.22 s by increasing the frame rate to the full refresh rate (17.86 kHz) of the DMD.

### Computer specifications

Our computer is equipped with a Microsoft Windows Server 2016 Standard operating system, an Intel Xeon CPU E5–2650 V4 @2.20 GHz, 64 Gb of DDR4 RAM memory, and a Nvidia GTX 1080 Ti GPU possessing 3584 CUDA cores running at 1.6 GHz and with 11 GB of GDDR5X memory running at 11 Gbps.

## Supplementary information

Supplementrary material

## Data Availability

Data supporting the findings in this study are available within the article and its supplementary information files and from the corresponding author upon reasonable request.
