# Supplementary material for: Optical orbital-angular-momentum-multiplexed data transmission under high scattering
Source: Light Sci Appl. 2019 Mar 6;8:27. doi: 10.1038/s41377-019-0140-3 (PMC6401086; doi:10.1038/s41377-019-0140-3)
Supplement: Supplementary file 1 — Supplementrary material [file 41377_2019_140_MOESM1_ESM.pdf]

Supplementary Information for

Optical Orbital-Angular-Momentum-Multiplexed Data Transmission Under High Scattering

Lei Gong[1,†,⋆], Qian Zhao[1,†], Hao Zhang[1], Xin-Yao Hu[1], Kun Huang[1,⋆], Jia-Miao Yang[2], and Yin-Mei Li[1,3,⋆]

[1]*Department of Optics and Optical Engineering, University of Science and Technology of China, Hefei 230026, China*

[2]*Andrew and Peggy Cherng Department of Medical Engineering, Department of Electrical Engineering, California Institute of Technology, Pasadena, California 91125, USA*

[3]*Hefei National Laboratory for Physical Sciences at the Microscale, University of Science and Technology of China, Hefei 230026, China*

[†]*These authors contributed equally to the work.*

⋆*Correspondence: leigong@ustc.edu.cn, huangk17@ustc.edu.cn,liyinmei@ustc.edu.cn*

Supplementary Figures

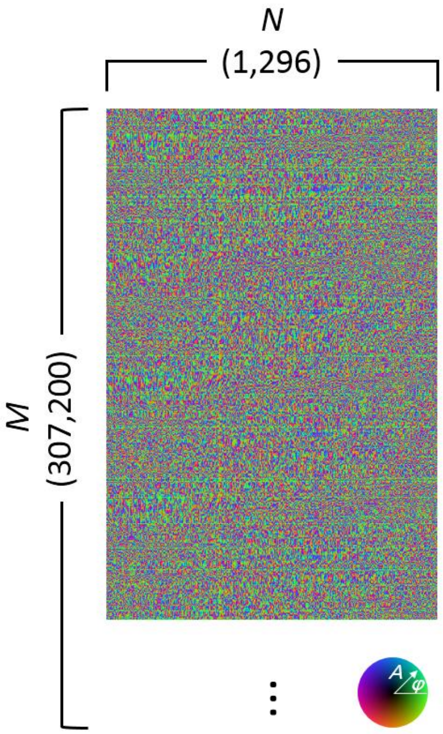

Supplementary Figure 1. Visualization of the calibrated transmission matrix (TM). We show here only part (1296×2000) of the measured TM, which is actually mapped onto the N = 1296 preset input bases and M = 307200 sampling points in the output plane. In the color circle, the A and φ symbols denote the normalized amplitude and phase, respectively.

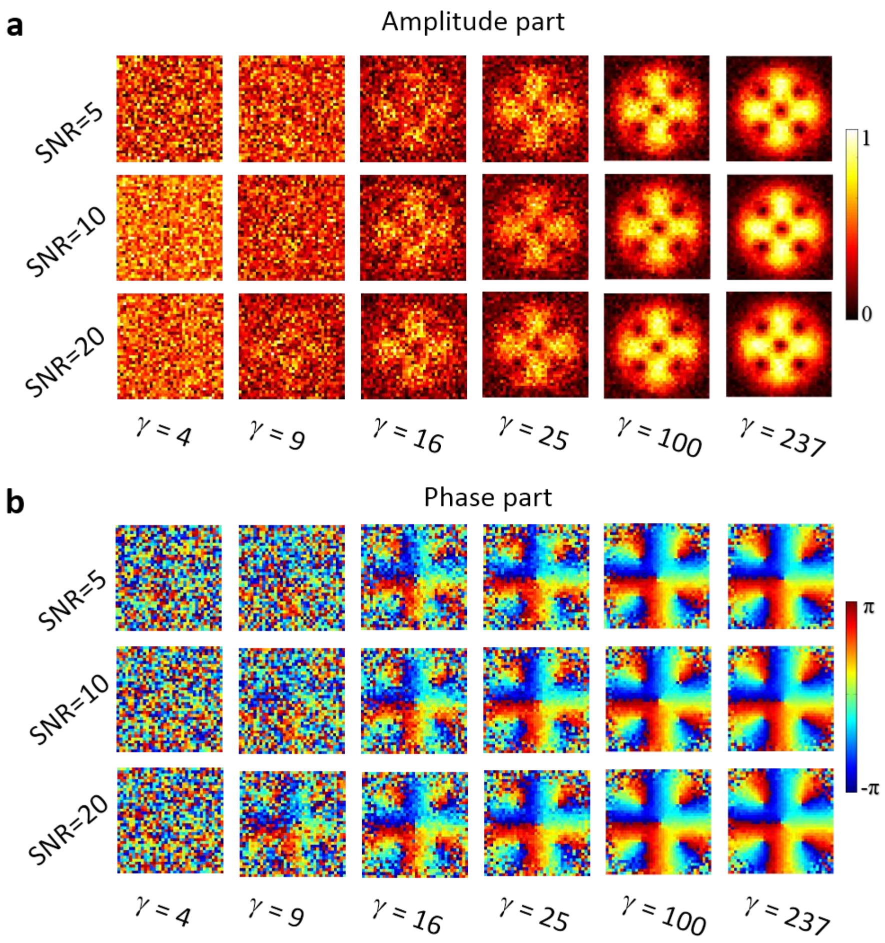

Supplementary Figure 2. The performance of field retrieval in terms of sampling rate (γ) and SNR. To investigate the effects of γ and SNR on the performance of field retrieval, we performed numerical simulations by adjusting γ and the SNR of added Gaussian noise. The retrieved fields including the amplitude (**a**) and phase (**b**) parts under different conditions are presented. The quality of the retrieved field becomes better as γ or SNR increases.

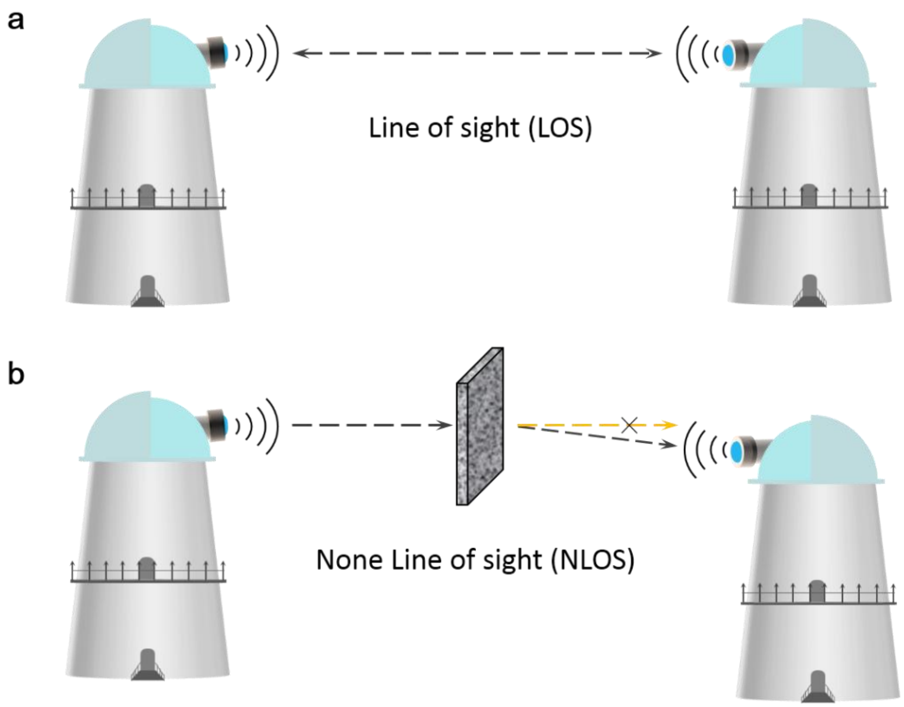

Supplementary Figure 3. Schematic illustration of optical wireless communication links. (**a**) Line-of-sight (LOS) connection and (**b**) none-line-of-sight (NLOS) connection.

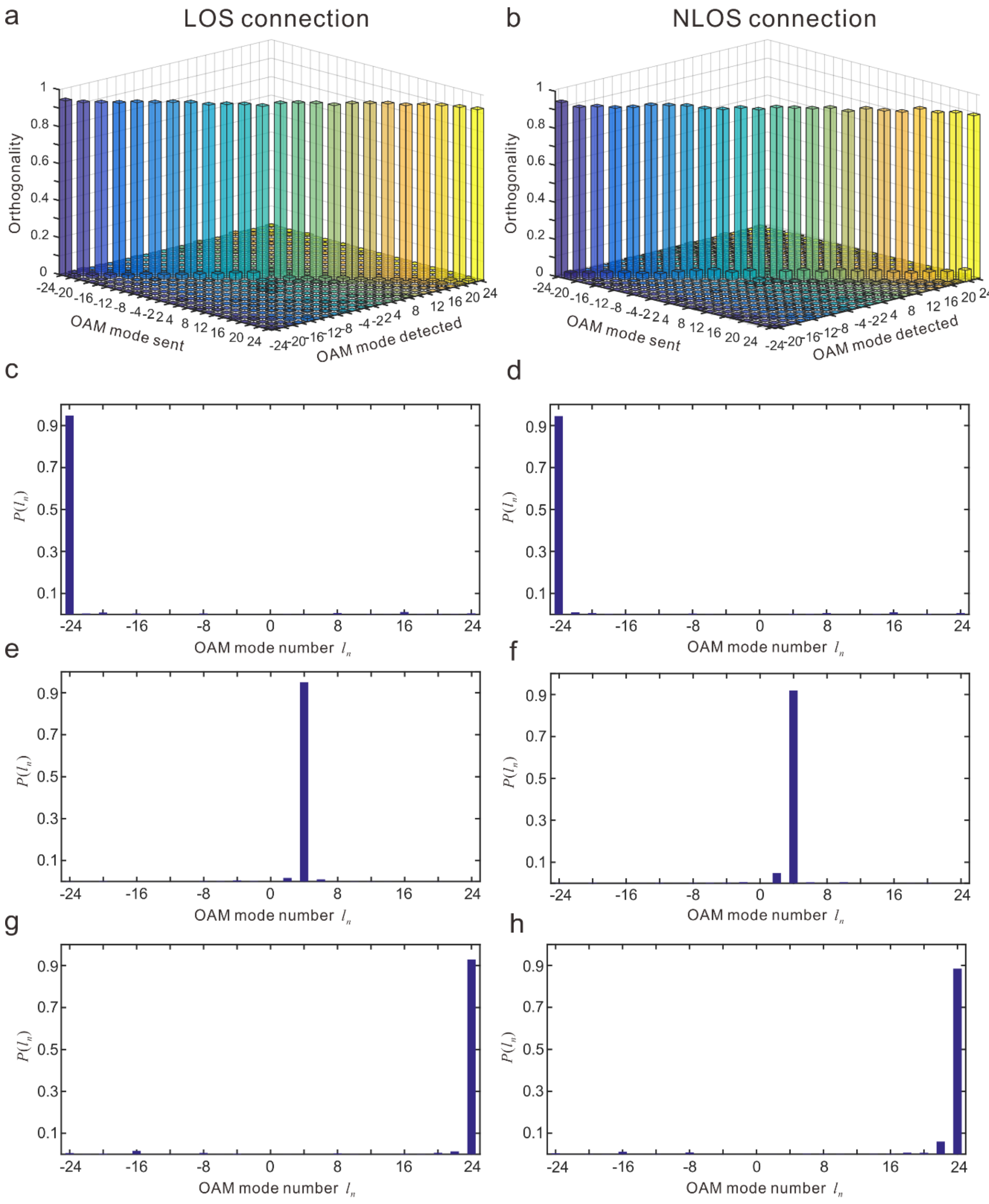

Supplementary Figure 4. Crosstalk matrix measured in different configurations. (**a**) The crosstalk matrix measured under on-axis detection. (**b**) The crosstalk matrix measured under off-axis detection. The on-axis (**c, e, g**) and off-axis (**d, f, h**) are the measured orthogonality relations of three OAM modes for comparison. The results indicate that SMART has a good tolerance to system misalignment.

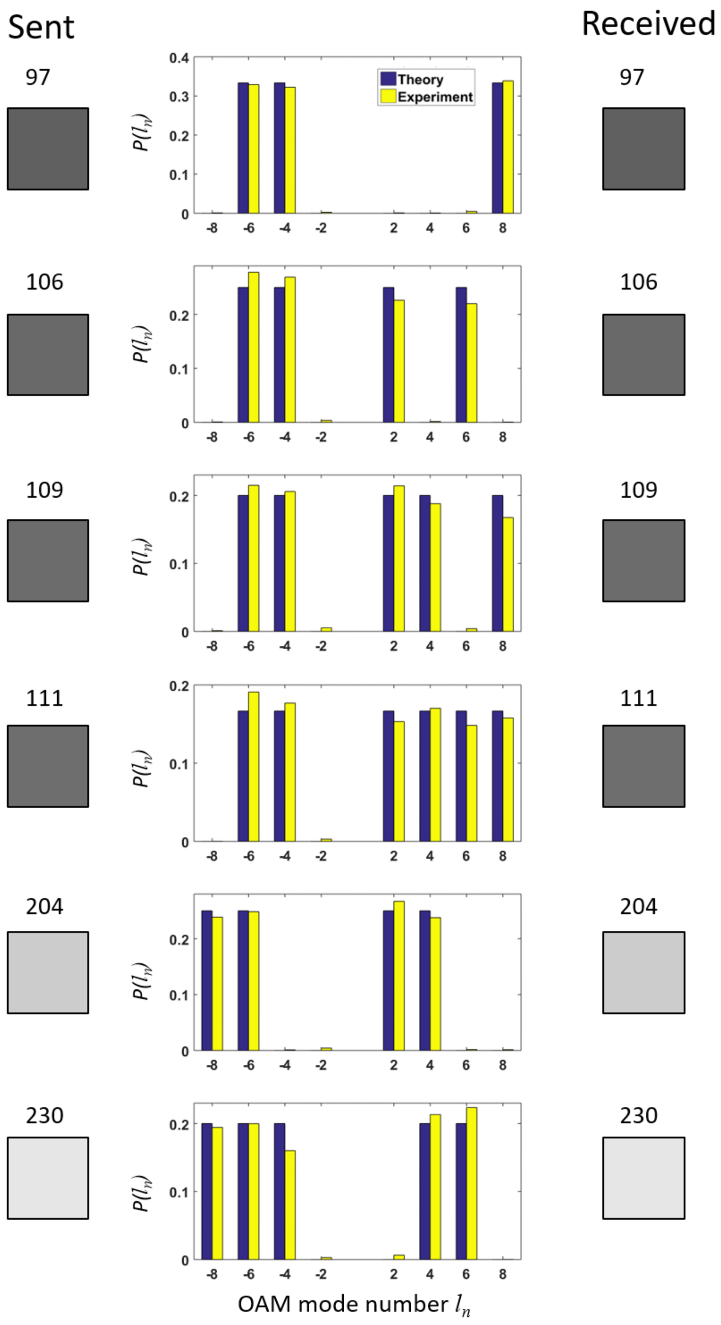

Supplementary Figure 5. Reconstructed OAM spectra for the received gray image. The sent and received gray levels are demonstrated for verifying the gray-scale encoding scheme. Corresponding measured and theoretical OAM spectra are also presented for further examination.

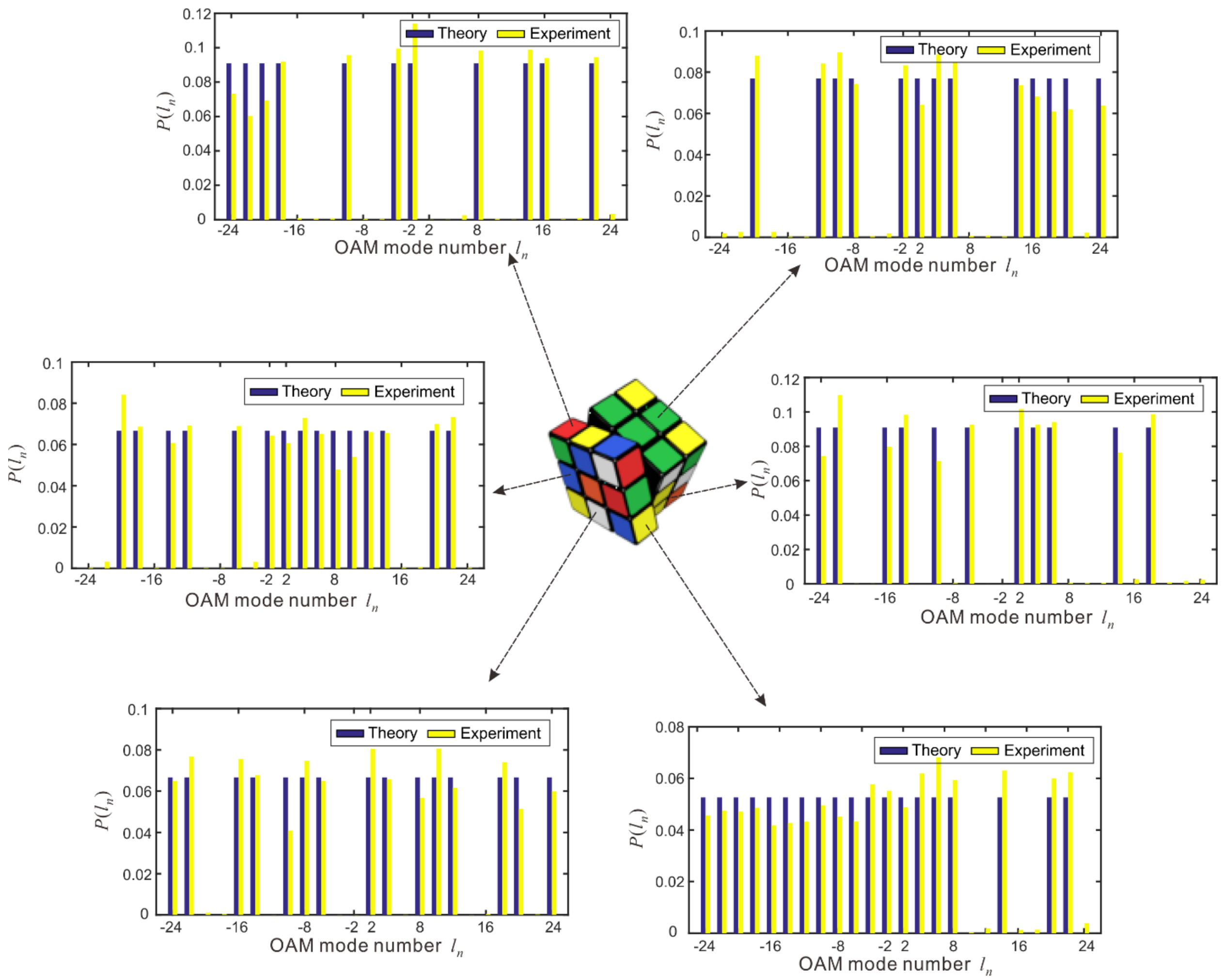

Supplementary Figure 6. Reconstructed OAM spectra for the received color image. The data-encoded light fields are uniquely identified and the information is decoded into its 24-bit form for the reconstruction of the image. Corresponding OAM spectra for the retrieved fields are presented for further examination.

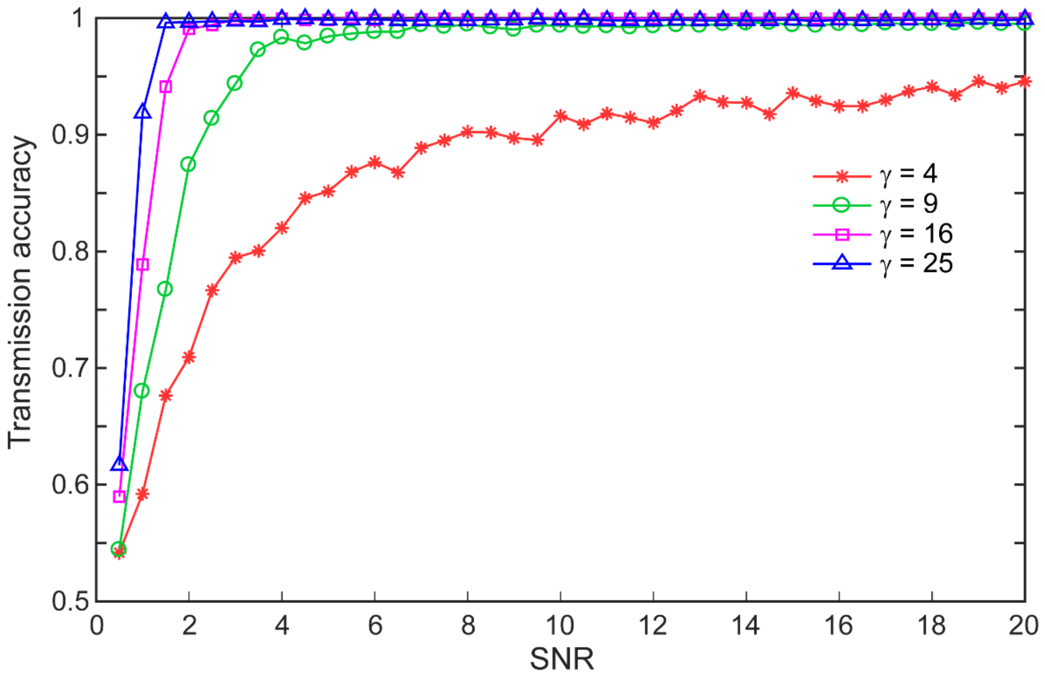

Supplementary Figure 7. Data transmission accuracy as a function of γ and SNR.

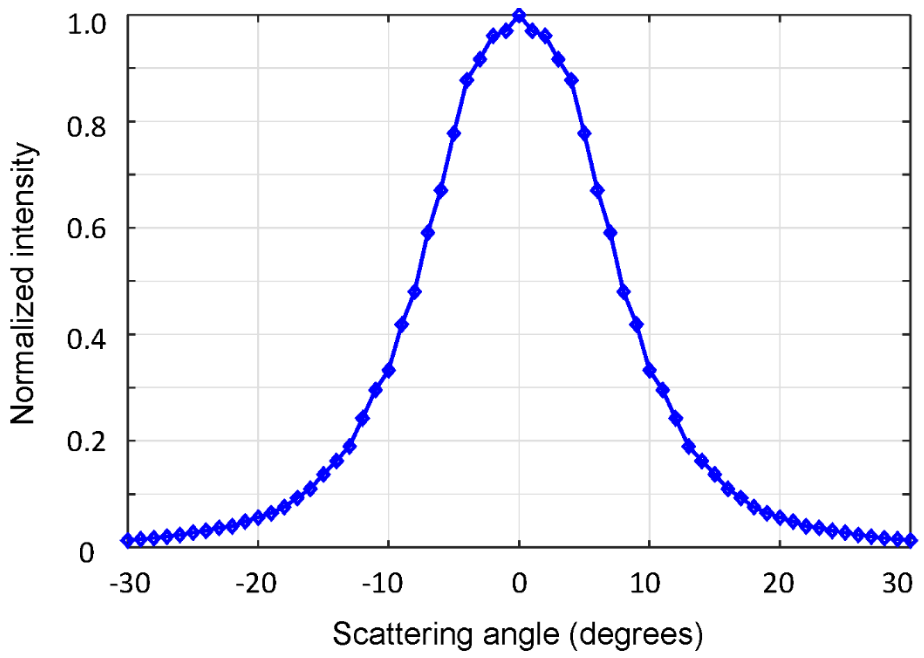

Supplementary Figure 8. Diffuser transmission as a function of the scattering angle. Raw data are provided by Thorlabs (https://www.thorlabschina.cn/newgrouppage9.cfm?objectgroup_id=1132).

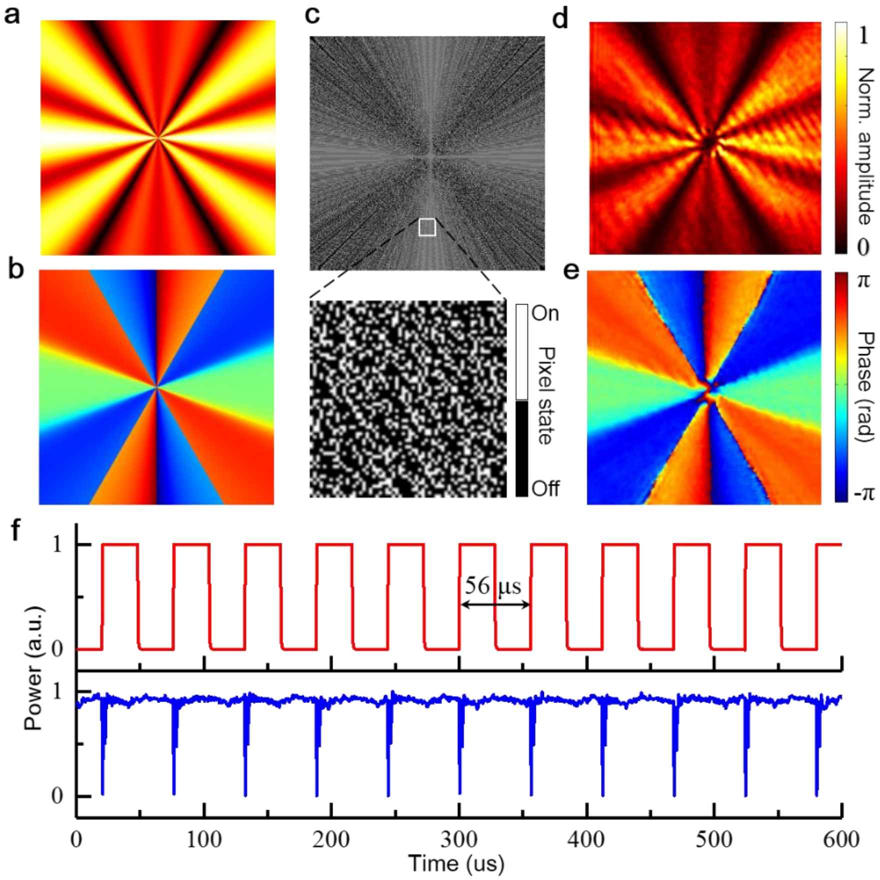

Supplementary Figure 9. Generation of the OAM beams by complex field encoding with a DMD. The desired amplitude (**a**) and phase (**b**) of the OAM mode at a given plane can be encoded with a binary hologram (**c**) using the super-pixel method. After the hologram is loaded onto the DMD, the desired field at z = 0 mm (**d, e**) is generated when the DMD is illuminated by a collimated laser beam. (**f**) The recorded waveform (blue) of dynamic switching among the generated OAM modes together with the DMD trigger waveform (red). The fastest switching speed is up to 17.86 kHz.

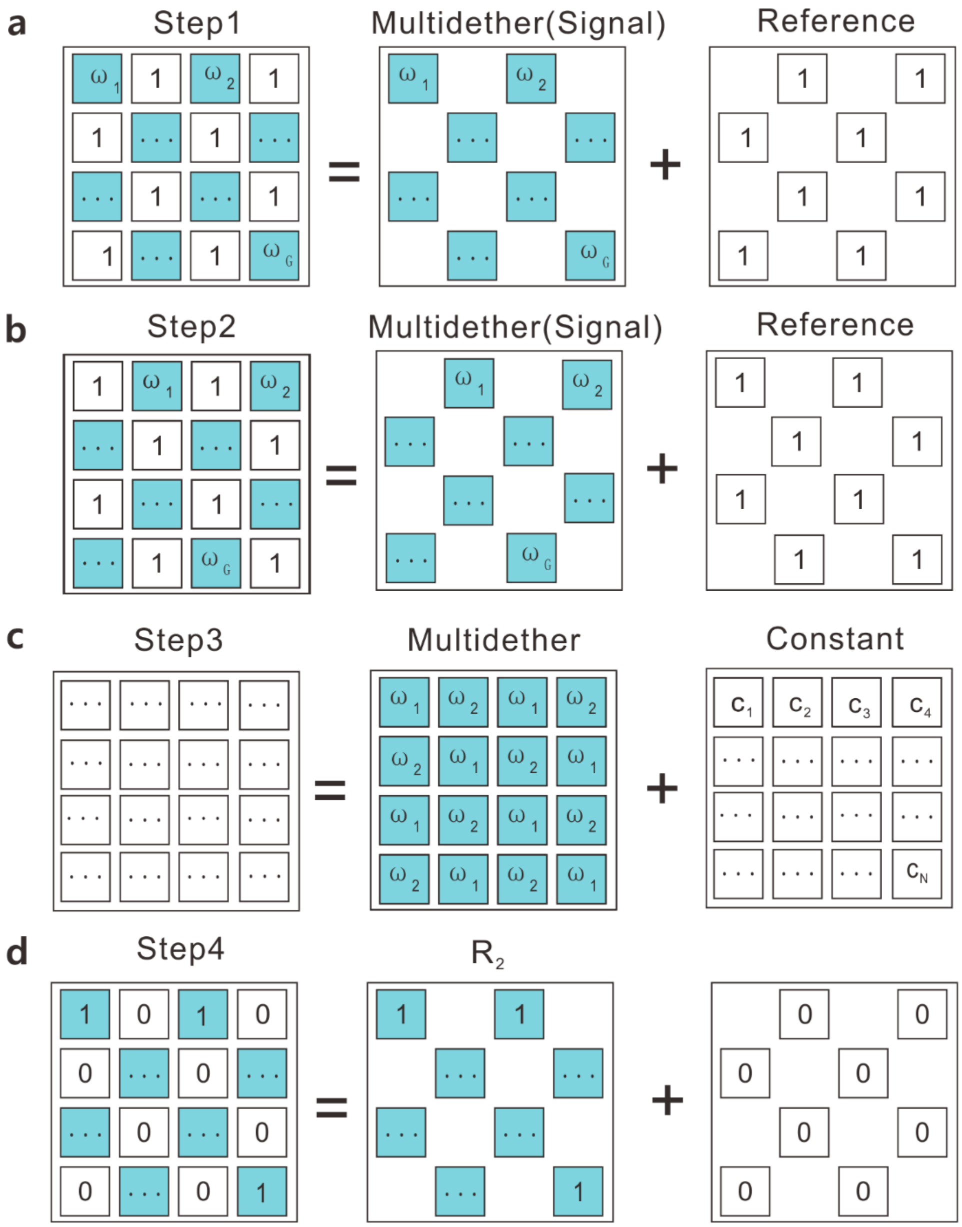

Supplementary Figure 10. Schematics for the experimental flow of TM calibration. (**a**) Group 1 measurement step. Individual pixels of Group 1 are modulated to act as signal part, while Group 2 stays still to provide a reference field. Required acquisitions are 3G. (**b**) Group 2 measurement step. Identical with (**a**) but the roles of the two groups are exchanged. Required acquisitions are 3(*N*-*G*). (**c**) Reference phase matching step. Each group rather than each pixel is modulated individually. Required acquisitions are 3. (**d**) Normalization of the reference intensity. One additional acquisition is needed after turning off the signal part. In total, 3*N*+7 acquisitions are adequate to completed the calibration process.

Supplementary Notes

Supplementary Note 1: Transmission matrix calibration algorithm

To achieve reference-free calibration with the same setup (Fig. 2a) used for optical transmission, we divide the DMD pixels into two groups that can be independently controlled to modulate the phase of the signal and reference light. Further, we introduce an algorithm that is developed from a parallel wavefront optimization method[1]. In the algorithm, each segment in the signal part is assigned with a distinct phase shifting frequency. The phase of each segment dithers at a unique frequency during the measuring process. This multi-dithering modulation allows us to parallelly access the complex TM elements via Fourier transformation.

Supplementary Fig. 9 presents the detailed implementations of the algorithm. Four steps are required to complete the calibration. For the first step (Supplementary Fig. 9a), one group modulates the signal field S_1 that consists of segments $k_1, k_2, \cdots, k_G$ modulating the impinging wavefront, where G is the number of segements in the signal group. Each segment in the signal group dithers at a unique frequency $\omega_1, \omega_2, \cdots, \omega_G$. While the other part consisting of pixels $k_{G+1}, k_{G+2}, \cdots, k_N$ stays still to offer a reference field R_1. Thus, the input field can be expressed as a sum of R_1 and S_1

$$x = R_1 + S_1 = \sum_{n=G+1}^{N} k_n + \sum_{n=1}^{G} k_n e^{i\omega_n t}. \tag{1}$$

After transmitted across the scattering channels, the light field can be described as:

$$y_m = T_m \cdot x = \sum_{n=G+1}^{N} t_{mn} k_n + \sum_{n=1}^{G} t_{mn} k_n e^{i\omega_n t}, \tag{2}$$

where y_m is the *m-th* output mode and T_m represents the *m-th* row of the transmission matrix.

In pratice, we can detect only the intensity map I_m of each output mode y_m, which reads

$$I_m = \left(T_m \cdot R_1\right)^2 + \sum_{p,q=1}^{G} t_{mp}^* t_{mq} e^{i\left(\omega_p - \omega_q\right)t} + \left(T_m \cdot R_1\right)^* \sum_{p=1}^{G} t_{mp} e^{i\omega_p t} + \left(T_m \cdot R_1\right) \sum_{p=1}^{G} t_{mp}^* e^{-i\omega_p t}. \tag{3}$$

The third term of the equation is the information related to TM. To decouple such term from the others, we define $\omega_p = \frac{G+p}{3G}\omega_0$ (ω_0 is the acquisition frequency of the detector) so that the ranges of ω_p and $\omega_q - \omega_p$, which are $\left(\frac{\omega_0}{3}, \frac{2\omega_0}{3}\right]$ and $\left(-\frac{\omega_0}{3}, \frac{\omega_0}{3}\right)$, cannot overlap with each other. Then, the information term $\left(T_m \cdot R_1\right)^* t_{mp}$ can be selectively extracted from the measured series of intensity maps by using phase shifting and Fourier transformation.

The second step involves the same procedure as the first one but with the roles of the two groups exchanged, as illustrated in Supplementary Fig. 9b. After that, two parts of the TM are obtained but with different reference terms

$$\begin{cases} \text{Group 1: } \left(T_m \cdot R_2\right)^* \left(t_{m1}, t_{m2}, \cdots, t_{mG}\right), \\ \text{Group 2: } \left(T_m \cdot R_1\right)^* \left(t_{m(G+1)}, t_{m(G+2)}, \cdots, t_{mN}\right). \end{cases} \tag{4}$$

It can be seen that our method fully uses the pixel resolution of the DMD, distinct from the calibration method the uses a fixed group as a reference[2]. In these two steps, at least 3*N* intensity measurements is required because the phase shifting is adopted.

To access the exact TM, however, we need to know the reference field. Therefore, the third step is to do the reference phase matching. To this end, the phase dithering of each group rather than each segment is performed in this step (Supplementary Fig. 9c). Actually, the segments of the two groups are assigned with two different phase-shifting frequencies ω_1 and ω_2, and an additional constant amplitude R_0 is added. Therefore, the designed input field for phase matching is

$$x = R_0 + R_1 e^{i\omega_1 t} + R_2 e^{i\omega_2 t}. \tag{5}$$

Obviously, Supplementary Eq. (5) is a special case of Supplementary Eq. (1) with $G = 2$. Thus, six measurements of speckle intensity maps are required to decouple the interference terms $\left(T_m R_0\right)^* \left(T_m R_1\right)$, $\left(T_m R_0\right)^* \left(T_m R_2\right)$, and $\left(T_m R_2\right)^* \left(T_m R_1\right)$ from the modulation frequencies ω_1, ω_2, and ω_1-ω_2, respectively. With the measured interference terms, we can perform the phase matching and further construct the TM as

$$\begin{cases} \text{Group 1: } (T_m \cdot R_2)^* (t_{m1}, t_{m2}, \cdots, t_{mG}), \\ \text{Group 2: } (T_m \cdot R_2)^* \left(t_{m(G+1)}, t_{m(G+2)}, \cdots, t_{mN}\right). \end{cases} \tag{6}$$

Furthermore, the last step involves normalization of the reference intensity, that is, measuring the modulus of the transmitted reference term $(T_m \cdot R_2)^*$. It can be achieved by taking one more image after turning off the signal part (Supplementary Fig. 9d). After this step, we finally obtain the calibrated TM multiplied by a phase factor of the complex conjugate of the reference field, which reads

$$\begin{cases} \text{Group 1: } \dfrac{(T_m \cdot R_2)^*}{\left|(T_m \cdot R_2)^*\right|} (t_{m1}, t_{m2}, \cdots, t_{mG}), \\ \text{Group 2: } \dfrac{(T_m \cdot R_2)^*}{\left|(T_m \cdot R_2)^*\right|} \left(t_{m(G+1)}, t_{m(G+2)}, \cdots, t_{mN}\right). \end{cases} \tag{7}$$

Note that, this phase term can be dismissed because it does not influence the field retrieval with the **Z**-matrix method.

Supplementary Note 2: Modal crosstalk analysis

The orthogonality relation between the OAM bases is obtained by forming inner product

$$c_{jl} = \int E_j^* E_l r dr d\phi, \tag{8}$$

where $E_l = A_l \exp(-il\phi)$ is the l-order OAM eigenstate. Actually, the orthogonality is quantified based on the intensity ratio of such term relative to the total intensity, which is calculated by

$$I_{jl} = \frac{\left|c_{jl}\right|^2}{\sum_j \left|c_{jl}\right|^2}, \tag{9}$$

where the total intensity is normalized such that $\sum_j \left|c_{jl}\right|^2 = 1$. The crosstalk is defined as[3]

$$crosstalk(dB) = 10 \log\left(I_{jl}\right). \tag{10}$$

For each sent OAM mode, the crosstalk between the retrieved field and all the OAM bases were measured, and thus the crosstalk matrix can be obtained.

Supplementary Note 3: Study the effects of γ and SNR on data transmission accuracy

To study the accuracy of OAM-multiplexed transmission, we performed numerical simulations under the different noise (SNR) and sampling rate (γ). To characterize it, OAM multiplexed fields of the $2^8 = 256$ possible combinations of 8 OAM bases were tested for every case. In the study, we added Gaussian noise to the transmission matrix, and measured the speckle intensity maps by adjusting γ and the SNR of noise. With SMART, the OAM-encoded data were extracted from the reconstructed OAM spectrum. By statistical analysis of the 256-times data transfer, the transmission accuracy, that is, 1–ER (error rate), was quantified for every case. Supplementary Fig. 7 presents the simulated results, verifying that SMART enables accurate data transfer under scattering. For example, a transmission accuracy better than 99.66% can be achieved when $\gamma \geqslant 25$ and SNR>2. Further increasing the SNR and sampling rate leads to the better performance in accuracy.

Supplementary Reference

1. Cui M. Parallel wavefront optimization method for focusing light through random scattering media. *Opt. Lett.* **36**, 870-872 (2011).

2. Popoff SM, Lerosey G, Carminati R, Fink M, Boccara AC, Gigan S. Measuring the Transmission Matrix in Optics: An Approach to the Study and Control of Light Propagation in Disordered Media. *Phys. Rev. Lett.* **104**, 100601 (2010).

3. Ren H, Li X, Zhang Q, Gu M. On-chip noninterference angular momentum multiplexing of broadband light. *Science* **352**, 805 (2016).
